# Fuzzy Sets in Dynamic Adaptation of Parameters of a Bee Colony Optimization for Controlling the Trajectory of an Autonomous Mobile Robot

**DOI:** 10.3390/s16091458

**Published:** 2016-09-09

**Authors:** Leticia Amador-Angulo, Olivia Mendoza, Juan R. Castro, Antonio Rodríguez-Díaz, Patricia Melin, Oscar Castillo

**Affiliations:** 1 Division of Graduate Studies and Research, Tijuana Institute of Technology, Tijuana 22414, Mexico; melin@tectijuana.edu.mx (P.M.); ocastillo@tectijuana.mx (O.C.); 2 Universidad Autónoma de Baja California, Tijuana 22390, Mexico; omendoza@uabc.edu.mx (O.M.); jrcastror@uabc.edu.mx (J.R.C.); ardiaz@uabc.edu.mx (A.R.-D.)

**Keywords:** bee colony optimization, fuzzy controller, fuzzy sets, uncertainty, dynamic adaptation, membership functions, perturbation, autonomous mobile robot

## Abstract

A hybrid approach composed by different types of fuzzy systems, such as the Type-1 Fuzzy Logic System (T1FLS), Interval Type-2 Fuzzy Logic System (IT2FLS) and Generalized Type-2 Fuzzy Logic System (GT2FLS) for the dynamic adaptation of the alpha and beta parameters of a Bee Colony Optimization (BCO) algorithm is presented. The objective of the work is to focus on the BCO technique to find the optimal distribution of the membership functions in the design of fuzzy controllers. We use BCO specifically for tuning membership functions of the fuzzy controller for trajectory stability in an autonomous mobile robot. We add two types of perturbations in the model for the Generalized Type-2 Fuzzy Logic System to better analyze its behavior under uncertainty and this shows better results when compared to the original BCO. We implemented various performance indices; ITAE, IAE, ISE, ITSE, RMSE and MSE to measure the performance of the controller. The experimental results show better performances using GT2FLS then by IT2FLS and T1FLS in the dynamic adaptation the parameters for the BCO algorithm.

## 1. Introduction

In 1965 Zadeh first proposed the concept of a fuzzy set (FS) [1]. His vision was set on giving more control over decision making, and with his fuzzy logic an immeasurable amount of decision- making situations could be easily modeled whereas hard logic, true or false, could not. This opened a new era in decision making with FSs that have been evolving since its initial days, first starting out with Type-1 Fuzzy Logic Systems (T1FLS), then coming into Interval Type-2 Fuzzy Logic Systems (IT2FLS) and finally arriving to the current state of advanced form of FS, Generalized Type-2 Fuzzy Logic Systems (GT2FLS).

In recent years, many works on control system stabilization have been published [2,3,4]. However, all these control design methods require the exact mathematical models of the physical systems, which may not be available in practice. On the other hand, fuzzy control has been successfully applied for solving many nonlinear control problems. Some works related in automatic control are [5,6,7,8,9,10]. Fuzzy logic or multi-valued logic is based on fuzzy set theory proposed in [1,11], which helps us in modeling knowledge, through the use of if-then rules. In Interval Type-2 fuzzy systems, the membership functions can now return a range of values, which vary depending on the uncertainty involved in not only the inputs, but also in the same membership functions [12,13]. In Generalized Type-2 fuzzy systems the uncertainty is depicted by a volume, and as such, being more capable of handling uncertainty in the system. As GT2FS research is still fairly new, not much has been done as of yet, some examples of advancements are shown in computing the centroid by means of the centroid-flow algorithm [14], definition of the footprint of uncertainty [15], enhanced type-reduction [16], conversion from IT2FS to GT2FS [3], computing with words for discrete GT2FS [17], and formation of GT2FS based on information granule numerical evidence [18].

In 1975 Mamdani and Assilian were the first to develop fuzzy logic controllers (FLCs) which, have been successfully applied in many real word applications [19], including cement kiln controllers, water treatment systems, and automatic train operation control systems, industrial tools such as robot arms, as well as in home appliances such as washing machines, vacuums, rice cookers, air conditioners, microwaves, and refrigerators. There are two main advantages of FLC over other nonlinear controllers. The first is the ability to incorporate the linguistic terms of input-output variables by using fuzzy membership functions. Second, it can more effectively handle the uncertainties in the inputs and state measurements [20,21]. Fuzzy controllers have the advantage that they can be adaptive when disturbances in the model or the plant are present. Usually fuzzy controllers are used to test the bio-inspired algorithms and observe their performance, for example; some works presented in this regard can be found in [22,23,24,25,26,27].

Optimization is a science which finds the best values of the parameters of a problem that may take under specified conditions. Optimization, in its most simple way, aims to obtain the relevant parameter values, which enable an objective function to generate a *minimum* or a *maximum* value depending on the problem. The objective function is the main component of an optimization problem [28].

The main idea of dynamic adjustment of parameters in algorithms or techniques involved with the optimization is of key interest to some researchers; for example, in [29] a fuzzy parameter adaptation in optimization of neural net training is presented, in [30] a fuzzy adaptive particle swarm optimization is presented, in [31] a nonlinear inertia weight variation for dynamic adaptation in particle swarm optimization is presented, in [32] an optimal design of fuzzy classification systems using PSO with dynamic parameter adaptation through fuzzy logic is presented, in [33] a differential evolution with dynamic adaptation of parameters of the optimization of fuzzy controller is presented. This is why we consider as the main contribution of this research, the use of fuzzy sets as a powerful technique to define the appropriate alpha and beta parameters in the BCO algorithm and thereby improve its performance for the solution of complex problems.

The Bee Colony Optimization (BCO) metaheuristic has been successfully applied to various engineering and management problems by Teodorović et al. [34,35,36]. It has been shown that the BCO algorithm is a good technique in solving complex problems, and to mention some current research in this regard; in [37] a bee colony optimization algorithm applied to job shop scheduling is presented by Chong et al., another work can be found in [38] where an efficient bee colony optimization algorithm for the traveling salesman problem using frequency-based pruning is presented by Wong et al., in [39] a bee colony optimization based-fuzzy logic-PID control design of electrolyzer for microgrid stabilization presented by Chaiyatham et al. and in [40] the design and development of an intelligent control by using bee colony optimization technique is presented by Tiacharoen et al.

This paper considers several experiments in the simulation of control problems with a type-1 fuzzy logic controller (T1FLC) and BCO for minimizing the error in the simulation of the trajectory controlling of an autonomous mobile robot. In this case, the fuzzy bee colony optimization shows better results based on error minimization when the generalized type-2 fuzzy logic system is used to adapt the *alpha* and *beta* parameters for BCO. We realized the comparison with the Original BCO and the Fuzzy BCO algorithm, using the three type fuzzy sets, for observing the behavior and the improvement that Fuzzy BCO provides when different levels of noise are applied in the model, in this case GT2FLS can handle the uncertainty to get a better stabilization of the autonomous mobile robot.

The rest of the paper is organized as follows. Section 2 describes some basic concepts of fuzzy sets and briefly describes generalized type-2 fuzzy logic systems. Section 3 shows fuzzy logic controllers. Section 4 outlines the problem statement that is used in the simulations. Section 5 describes the traditional bee colony optimization (BCO) algorithm and fuzzy BCO. Section 6 shows the simulation results with the dynamic adaptation in the parameters of BCO with different fuzzy systems. Section 7 shows the discussion of results. Finally, Section 8 offers some conclusions of this work.

## 2. Fuzzy Sets

### 2.1. Type-1 Fuzzy Logic System

A type-1 fuzzy set in the universe X is characterized by a membership function $\mu_{A}\left(x\right)$ taking values on the interval [0, 1] and can be represented as a set of ordered pairs of an element and the membership degree of an element to the set and are defined by the following Equation (1) [1,4,20,21,41,42]:

(1) $$A=\{(x,\mu_{A}\left(x\right))\;|\;x\;\in \;X\}$$

where $\mu_{A}:X\to \left[0,1\right].$

**Figure 1:**
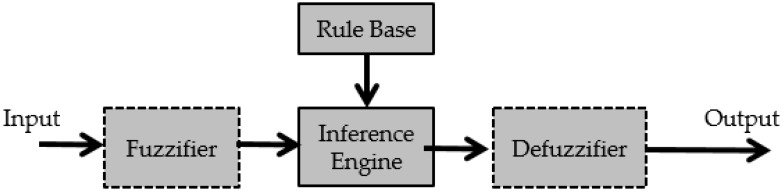
Type-1 fuzzy logic system.

In this definition $\mu_{A}\left(x\right)$ represents the membership degree of the element x ∈X to the set A. In this work we are going the use the following notation: $A\left(x\right)={\;\mu }_{A}\left(x\right)$ for all x ∈X. Figure 1 shows the Type-1 Fuzzy Logic System.

### 2.2. Interval Type-2 Fuzzy Logic System

Based on Zadeh’s ideas, Mendel et al. presented the mathematical definition of a type-2 fuzzy set, as follows [20,21].

**Figure 2:**
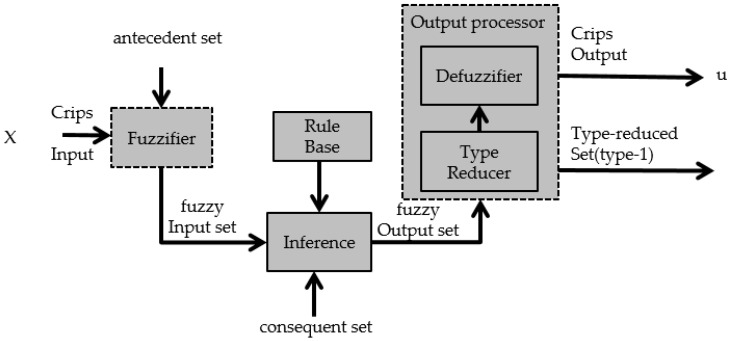
Interval Type-2 fuzzy logic system.

An Interval Type-2 Fuzzy Set $\tilde{A}$, denoted by ${\underline{\mu }}_{\tilde{A}}(x)$ and ${\bar{\mu }}_{\tilde{A}}(x)$ is represented by the lower and upper membership functions of $\mu_{\overset{ˇ}{A}}\left(x\right)$. Where x ∈X. In this case, Equation (2) shows the definition of an IT2FS [43,44,45,46,47,48,49]:

(2) $$\tilde{A}=\;\left\{\left(\left(x,u\right),1\right)|\;\forall x\in X,\;\forall u\in {\;J}_{x}\;\subseteq \;\left[0,1\right]\right\}$$

where X is the primary domain, Jx is the secondary domain. All secondary degrees $\left(\mu_{\tilde{A}}\left(x,u\right)\right)$ are equal to 1. Figure 2 shows the representation of an Interval Type-2 Fuzzy Logic System.

The output processor includes a type-reducer and defuzzifier that generates a type-1 fuzzy set output (from the type-reducer) or a crisp number (from the defuzzifier) [46,49]. An Interval type-2 FLS is also characterized by IF-THEN rules, but their fuzzy sets are now of interval type-2 form. The Type-2 Fuzzy Set can be used when circumstances are too uncertain to determine exact membership degrees, as is the case with the membership functions in a fuzzy controller that can take different values and we want to find the distribution of membership functions that show better results in the stability of the fuzzy controller [50,51].

### 2.3. Generalized Type-2 Fuzzy Logic System

**Figure 3:**
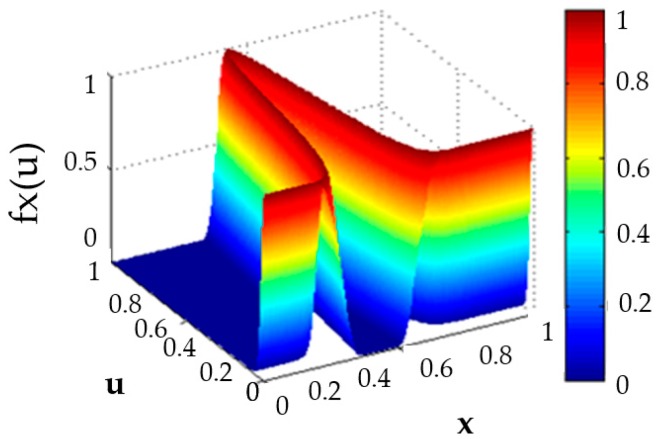
Generalized Type-2 membership function.

**Figure 4:**
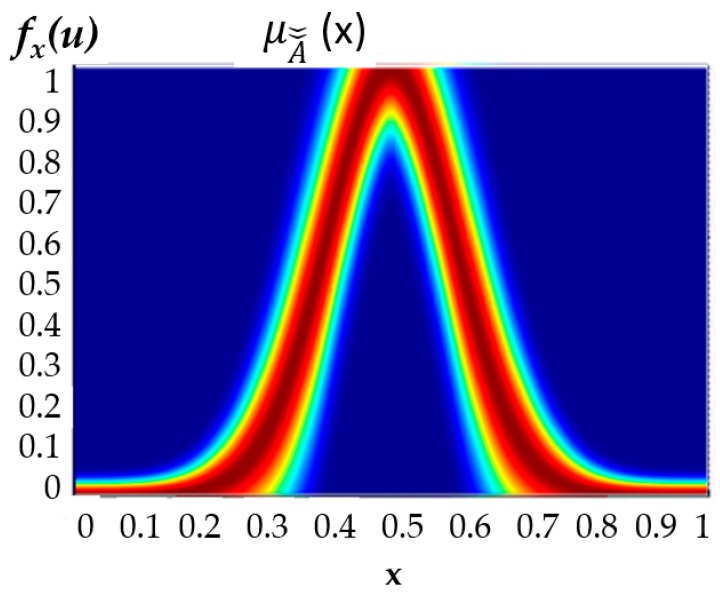
FOU of the Generalized Type-2 membership function.

With GT2FLS the logic is generally the same as for T1FLS and IT2FLS, but their operations are somewhat different, due to the nature of GT2FS [51,52]. Generalized Type-2 Fuzzy Sets are defined by the following Equation (3):

(3) $$\tilde{\tilde{A}}=\;\left\{\left(\left(x,u\right),\mu_{\tilde{A}}\left(x,u\right)\right)|\;\forall x\in X,\;\forall u\in {\;J}_{x}\;\subseteq \;\left[0,1\right]\right\}$$

where $J_{x}\subseteq \left[0,\;1\right]$, x is the partition of the primary membership function, and u is the partition of the secondary membership function. In Figure 3 we can find a representation of a generalized type-2 membership function, and in Figure 4, the footprint of uncertainty (FOU) is illustrated, which is associated with the third dimension and allows a better modeling of real world uncertainty.

**Figure 5:**
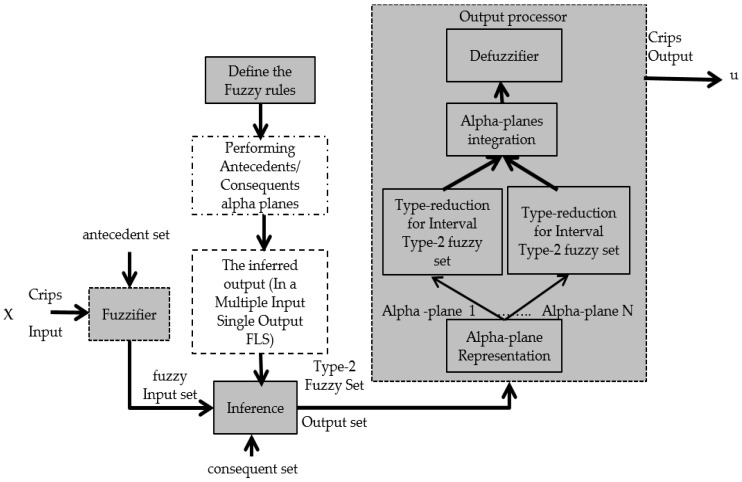
Generalized Type-2 fuzzy logic system.

It must be noted that there is a small difference in notation when compared with Type-1 and Interval Type-2, this is, T1FS and IT2FS use the notation μ(x), but GT2FS uses $f_{x}\left(u\right)$, in the vertical axis, and this is due to the complexity involved in GT2FLS in comparison with the others, as well as how GT2FLS has been described in the literature [53]. Figure 5 shows the representation of a Generalized Type-2 Fuzzy Logic System.

#### 2.3.1. Fuzzification

The fuzzifier maps crisp inputs into generalized type-2 fuzzy sets to process within the FLC. In this paper, we will focus on the type-2 singleton fuzzifier as it is fast to compute and, thus, suitable for the generalized type-2 FLC real-time operation. Singleton fuzzification maps the crisp input into a fuzzy set, which has a single point of nonzero membership. Hence, the singleton fuzzifier maps the crisp input $x_{p}^{\prime }$ into a type-2 fuzzy singleton, whose MF is $\mu_{{\tilde{A}}_{p}}\left(x_{p}\right)=1/1$ for $x_{p}=\;x_{p}^{\prime }$ and $\mu_{{\tilde{A}}_{p}}\left(x_{p}\right)=0$ for all $x_{p}\neq \;x_{p}^{\prime }$ for all p=1, 2, …, P, where *P* is the number of FLS inputs [54].

#### 2.3.2. Inference

Once the input and output variables are defined, with their respective membership functions, the inference process is performed in the system, and for this the following steps are needed:

Define the Fuzzy Rules: The structure of the rules in the generalized Type-2 FLS is the standard Mamdani-type FLS rule structure used in the Type-1 FLS and an interval Type-2 FLS, but in this paper, we assume that the antecedents and the consequents sets are represented by generalized Type-2 fuzzy sets. So for a Type-2 FLS with p inputs $x_{1}\;\epsilon {\;X}_{1},\;\ldots ,{\;x}_{p}\;\in {\;X}_{P}$ and one output y∈Y, Multiple Input Single Output (MISO), if we assume there are M rules, the kth rule in the generalized type-2 FLS can be written as follows [52,53,54]:

(4) $$R^{k}:\mathrm{IF}\;x_{1}\;\mathrm{is}\;{\tilde{F}}_{1}^{k}\;\mathrm{and}\ldots \mathrm{and}\;x_{p}\;\mathrm{is}\;{\tilde{F}}_{p}^{k},\;\mathrm{THEN}\;y\;\mathrm{is}\;{\tilde{G}}^{k}$$

The inference of a GT2FLS can be simplified into two main operations, meet and join, as shown in Equations (5) and (6), respectively

(5) $$\mu_{\tilde{A}}(x,u)⊔\mu_{\tilde{B}}(x,w)=\{(v,f_{x}(u)\tilde{*}f_{x}(w))\;|\;v\in u\vee w,\;u\in J_{x}^{u}\subseteq [0,1],\;w\in J_{x}^{w}\subseteq [0,1]\}$$

(6) $$\mu_{\tilde{A}}(x,u)⊓\mu_{\tilde{B}}(x,w)=\{(v,f_{x}(u)\tilde{*}f_{x}(w))\;|\;v\in u^{\wedge }w,\;u\in J_{x}^{u}\subseteq [0,1],\;w\in J_{x}^{w}\subseteq [0,1]\}$$

#### 2.3.3. *α*-Planes Representation

**Figure 6:**
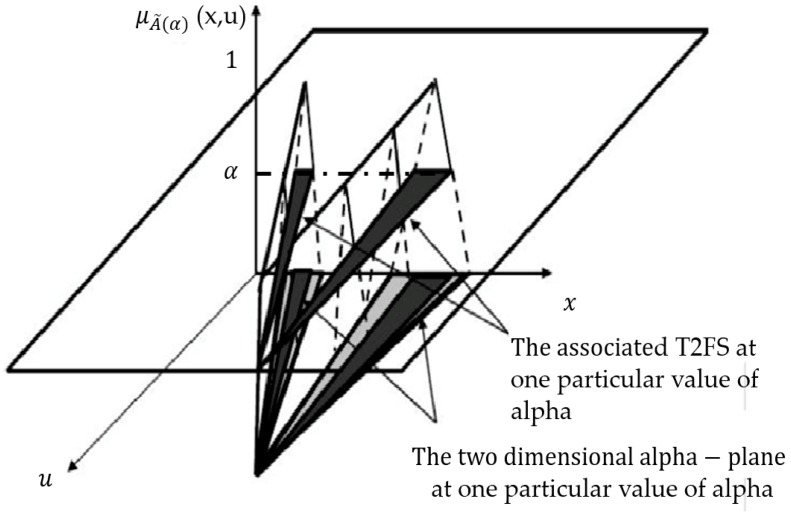
An example of the associated type-2 fuzzy set for the alpha-plane.

The α-plane for a generalized T2 FLS, in this case $\tilde{A}$, is denoted by Ã*α*, and it is the union of all primary membership functions of Ã, which secondary membership degrees are higher or equal to α (0 ≤ *α*≤ 1) [45,46]. The equation of an alpha plane is represented by Equation (7). In Figure 6 the representation of an alpha plane is illustrated [55,56,57]:

(7) $${Ã}_{\alpha }=\;\left\{\left(x,u\right),\mu_{Ã}\left(x,u\right)\geq \alpha |\forall x\in X,\forall u\in J_{X\;}\subseteq \left[0,1\right]\right\}$$

#### 2.3.4. Type Reduction

Type reduction is performed by applying the type reductor of the Karnik and Mendel algorithm [43,48], and this reduction is given by the following Equations (8) and (9):

(8) $$y_{\propto }^{l}\left(x^{\prime }\right)=\;\frac{\sum_{k\;=\;1}^{L}{\bar{\Omega }}_{\propto }^{k}\left(x\prime \right){\bar{y}}_{l}^{i}+\;\sum_{j\;=\;L\;+\;1}^{M}{\underline{\Omega }}_{\propto }^{j}\left(x\prime \right){\bar{y}}_{l}^{j}}{\sum_{k\;=\;1}^{L}{\bar{\Omega }}_{\propto }^{k}\left(x\prime \right)+\sum_{j\;=\;L\;+\;1}^{M}{\underline{\Omega }}_{\propto }^{j}\left(x\prime \right)}$$

(9) $$y_{\propto }^{r}\left(x^{\prime }\right)=\;\frac{\sum_{k\;=\;1}^{R}{\underline{\Omega }}_{\propto }^{k}\left(x\prime \right){\bar{y}}_{r\;}^{k}+\;\sum_{k\;=\;R\;+\;1}^{M}{\bar{\Omega }}_{\propto }^{k}\left(x\prime \right){\bar{y}}_{r\;}^{k}}{\sum_{i\;=\;1}^{R}{\underline{\Omega }}_{\propto }^{i}\left(x\prime \right)+\sum_{i\;=\;R\;+\;1}^{M}{\bar{\Omega }}_{\propto }^{i}\left(x\prime \right)}$$

The results of the alpha planes are integrated by the following Equations (10) and (11) [55,56]:

(10) $${ŷ}_{j}^{l}\left(x^{\prime }\right)=\frac{\sum_{i\;=\;1}^{N}\propto_{i}^{\propto_{i}}y_{j}^{l}\left(x^{\prime }\right)}{\sum_{i\;=\;1}^{N}\propto_{i}}$$

(11) $${ŷ}_{j}^{r}\left(x^{\prime }\right)=\;\frac{\sum_{i\;=\;1}^{N}\propto_{i}^{\propto_{i}}y_{j}^{r}\left(x^{\prime }\right)}{\sum_{i\;=\;1}^{N}\propto_{i}}$$

#### 2.3.5. Defuzzification

After realizing the type reduction and integrating the results of all the alpha planes, the defuzzification is performed by using the average of $y_{l}$ and $y_{r}$, to obtain the defuzzified output of a generalized type-2 non-singleton FLS [58,59]:

(12) $${ŷ}_{j}\left(x^{\prime }\right)=\frac{{ŷ}_{j}^{l}\left(x^{\prime }\right)+{ŷ}_{j}^{r}\left(x^{\prime }\right)}{2}$$

## 3. Fuzzy Controllers

**Figure 7:**
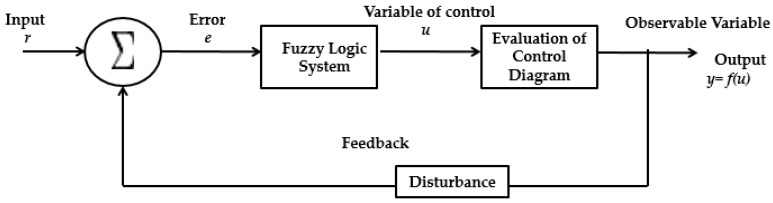
General representation of a FLC.

Early, a Fuzzy Logic Controller (FLC) was designed only using type-1 fuzzy sets in representing the input-output uncertainties. However, these are uncertainties in the meaning of words in the antecedents and consequents of the rules, the histogram values of the consequents extracted from a group of experts, and the noisy data as well as measurements [2,3,4,33,41,42,59]. Type-1 fuzzy sets have limited ability to handle such uncertainties because they apply crisp membership functions. In Figure 7 the generic representation of the FLC is illustrated.

## 4. Problem Statement

### 4.1. General Description

**Figure 8:**
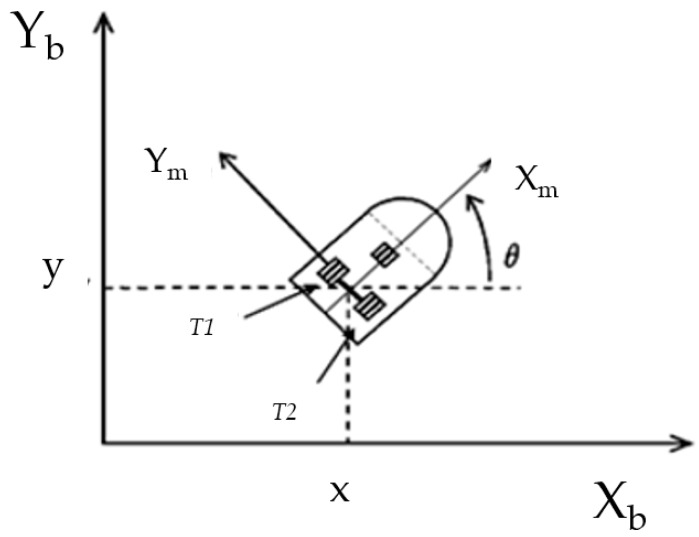
Mobile robot model.

The model which is used is of a unicycle mobile robot [2,3,4,42,59], consisting of two driving wheels located on the same axis and a front free wheel. Figure 8 shows a graphical description of the robot model.

The robot model assumes that the motion of the free wheel can be ignored in its dynamics, as shown in Equations (13) and (14):

(13) $$M\left(q\right)\dot{v}+C\left(q,\dot{q}\right)v+Dv=\tau +P\left(t\right)$$

where:

$q={\left(x,y,\theta \right)}^{T}$ is the vector of the configuration coordinates,
$\upsilon ={\left(v,w\right)}^{T}$ is the vector of velocities,
$\tau =\left(\tau_{1},\tau_{2}\right)$ is the vector of torques applied to the wheels of the robot where $\tau_{1}$ and $\tau_{2}$ denote the torques of the right and left wheel, respectively.
$P\in R^{2}$ is the uniformly bounded disturbance vector,
$M\left(q\right)\in R^{2\times 2}$ is the positive-definite inertia matrix,
$C\left(q,\dot{q}\right)\vartheta$ is the vector of centripetal and Coriolis forces, and
$D\in R^{2\times 2}$ is a diagonal positive-definite damping matrix.

The kinematic system is represented by Equation (14):

(14) $$\dot{q}=\underset{J\left(q\right)}{\underset{︸}{\left[\begin{array}{c} \cos\;\theta \\ \sin\;\theta \\ 0 \end{array}\begin{array}{c} 0\\ 0\\ 1 \end{array}\right]}}\underset{\upsilon }{\underset{︸}{\left[\begin{array}{c} v\\ w \end{array}\right]}}$$

where:

(*x,y*) is the position in the X − Y (world) reference frame,
*θ* is the angle between the heading direction and the *x*-axis,
*v* and *w* are the linear and angular velocities.

Furthermore, Equation (15) shows the non-holonomic constraint which this system has, which corresponds to a no-slip wheel condition preventing the robot from moving sideways:

(15) $$\dot{y}\cos\theta -\dot{x}\sin\theta =0$$

The system fails to meet Brockett’s necessary condition for feedback stabilization, which implies that no continuous static state-feedback controller exists that can stabilize the closed-loop system around the equilibrium point.

### 4.2. Characteristics of the Fuzzy Controller

**Figure 9:**
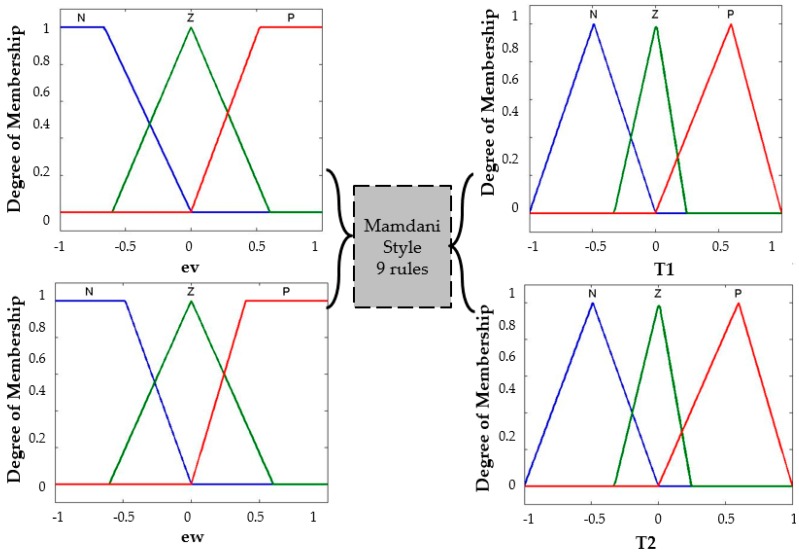
Characteristics of the Type-1 FLC of the mobile robot controller.

The main problem to study is controlling the stability of the trajectory in a mobile robot. The Membership functions are for the two inputs to the fuzzy system: the first is called *ev* (angular velocity), which has three membership functions with linguistic values of *N (Negative)*, *Z (Zero)* and *P (Positive).* The second input variable is called *ew* (linear velocity) with three membership functions with the same linguistic values. The type-1 fuzzy logic controller has two outputs called *T1* (Torque 1), and *T2* (Torque 2), which are composed of three triangular membership functions with the following linguistic values, respectively: *N (Negative)*, *Z (Zero)*, *P (Positive),* and in Figure 9 we show the representation of the input and output variables.

**Figure 10:**
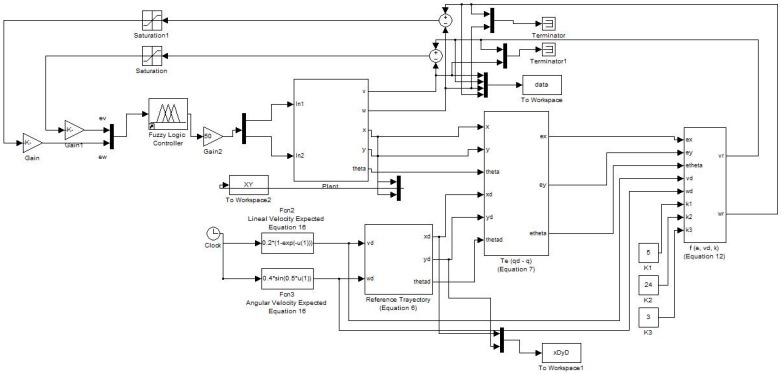
Fuzzy controller of the autonomous mobile robot.

The knowledge about the problem provides us with nine fuzzy rules for control. The combination of the rules is shown in Table 1 and Figure 10 shows the model of the Fuzzy Logic Controller.

The rules are selected based on the following references [3,4,59]. We choose the initial FIS with the nine rules set out in Table 1. For example, the third rule; when the angular velocity (*ev*) is Negative and linear velocity (*ew*) is Positive then the output Torque 1 (T1—Wheel right) is Negative (it indicates no movement) and Torque 2 (T2—Wheel left) is Positive (it indicates movement). Each torque has independent functions with a direct relationship that depending on the *ev* and *ew* values.

## 5. Bee Colony Optimization

The Bee Colony Optimization algorithm has recently received many improvements and applications. The BCO algorithm mimics the food foraging behavior of swarms of honey bees [35]. Honey bees use several mechanisms like the waggle dance to optimally locate a food source and search for new ones. It is a very simple, robust and population based stochastic optimization algorithm [36].

### 5.1. Traditional Bee Colony Optimization Algorithm

The communication between individual insects in a colony of social insects has been well known. The BCO is inspired by the bees´ behavior in nature. The basic idea behind the BCO is to create the multi agent system (colony of artificial bees) capable to successfully solve difficult combinatorial optimization problems. The artificial bee colony behaves partially alike, and partially differently from bee colonies in nature [34,35,36,37,38,39,40,42]. The algorithm parameters, whose values need to be set prior the algorithm execution are; B indicates the number of bees in the hive and NC indicates the number of constructive moves during one forward pass. In the beginning of the search, all the bees are in the hive.

The basic steps of the BCO algorithm are shown in Table 2. The BCO algorithm is based on Equations (16)–(19):

(16) $$P_{ij,n}=\frac{{\left[\rho_{ij,n}\right]}^{\alpha }.{\left[\frac{1}{d_{ij}}\right]}^{\beta }}{\sum_{j\;\in \;A_{i,n}}{\left[\rho_{ij,n}\right]}^{\alpha }.{\left[\frac{1}{d_{ij}}\right]}^{\beta }}$$

(17) $$D_{i}=K.\frac{Pf_{i}}{Pf_{colony}}$$

(18) $$Pf_{i}=\frac{1}{L_{I}},L_{i}=\mathrm{Tour}\;\mathrm{Length}$$

(19) $$Pf_{colony}=\frac{1}{N_{Bee}}\sum_{i\;=\;1}^{N_{Bee}}Pf_{i}$$

Equation (16) indicates the probability of a bee k located on a node *i* selects the next node denoted by *j*, where, *Nk_i_* is the set of feasible nodes (in a neighborhood) connected to node *i* with respect to bee k, and ρ*_ij_* is the probability to visit the following node. Note that the *β* is inversely proportional to the distance of the node; *d_ij_* represents the distance of node *i* until node *j*, for this algorithm indicate the total the dance that a bee have in this moment. Finally, α is a binary variable that is used to find better solutions in the algorithm. Equation (17) represents that a waggle dance will last for a certain duration, determined by a linear function, where K denotes the waggle dance scaling factor, *Pf_i_* denotes the profitability scores of bee *i* as defined in Equation (18) and *Pf_colony_* denotes the bee colony’s average profitability as in Equation (19) and is updated after each bee completes its tour. For this research the waggle dance is represented by the mean square error (MSE), which it is the representation of the fitness function in the fuzzy control analyzed, for each iteration in BCO algorithm a MSE is found, the main objective is to find the smallest error can stabilize the trajectory of an autonomous mobile robot. In the BCO algorithm, a bee represents the values of the distribution of the membership functions. The design of the T1FLS for the mobile robot controller has trapezoidal and triangular membership functions in the inputs and outputs (see Figure 9), giving a total of 40 values.

### 5.2. Fuzzy Bee Colony Optimization Algorithm

In the BCO algorithm the waggle dance represents the intensity with which a bee finds a possible good solution. If the intensity of the waggle dance is large this means that the solution found by the bee is the best of all the population [60]. For this work the waggle dance is represented by the mean square error (MSE) that all models find once the simulation in the iteration of the algorithm is done [41,42]. For measuring the iterations of the algorithm, it was decided to use the percentage of iterations as a variable, i.e., when starting the algorithm the iterations will be considered ‘‘low’’, and when the iterations are completed it will be considered ‘‘high’’ or close to 100%. We represent this idea using Equation (20) [32]:

(20) $$\mathrm{Iteration}=\frac{\mathrm{Current Iteration}}{\mathrm{Maximum of Iterations}}$$

The diversity measure is defined by Equation (21), which measures the degree of dispersion of the bees, i.e., when the bees are closer together; there is less diversity as well as when bees are separated then the diversity is higher. As the reader will realize the equation of diversity can be considered as the average of the Euclidean distances between each bee and the best bee. The main objective of using diversity is to provide the BCO algorithm with the ability to avoid getting trapped in local minimum; this is because the diversity represents the situation when the bees are not separated in the search space. This behavior is controlled with the rules that were designed with the Generalized Type-2 Fuzzy Logic System [32]:

(21) $$Diversity(S(t))=\frac{1}{n_{s}}\sum_{i\;=\;1}^{n_{x}}\sqrt{X_{ij}(t)-{\bar{X}}_{j}(t){)}^{2}}$$

where *t* indicates the current iteration, $n_{s}$ indicates the size of the population, *i* represents the bee, $n_{x}$ indicates the number of solutions, *j* represents the next solution in the space search, $X_{ij}$ indicates solution *j* of the bee *i*, finally, $\bar{X}$*_j_* represents solution *j* of the best bee in the space search.

The fitness function in the BCO algorithm is calculated with the Mean Square Error and is shown in Equation (22). For each Follower Bee for N cycles, the Type-1 FLS design for the BCO algorithm is evaluated and the objective is to minimize the error:

(22) $$MSE=\frac{1}{n}\sum_{i\;=\;1}^{n}({\bar{Y}}_{i}-Y_{i}{)}^{2}$$

**Figure 11:**
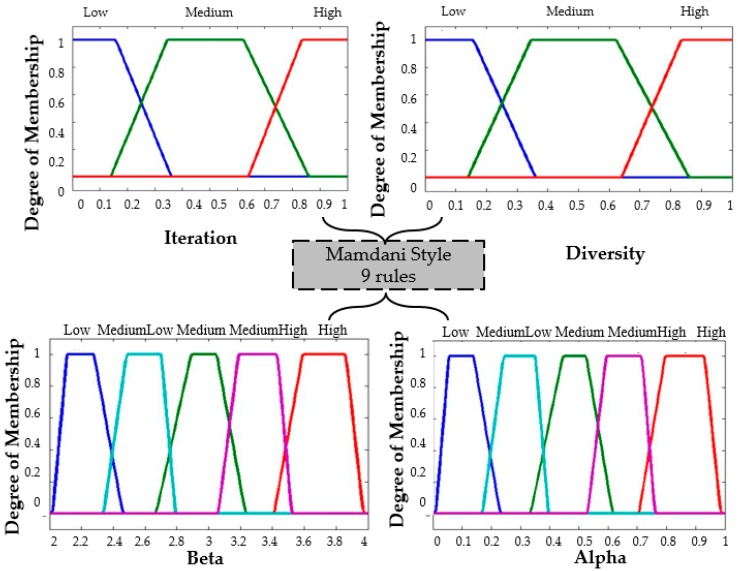
Fuzzy BCO with T1FLS.

**Figure 12:**
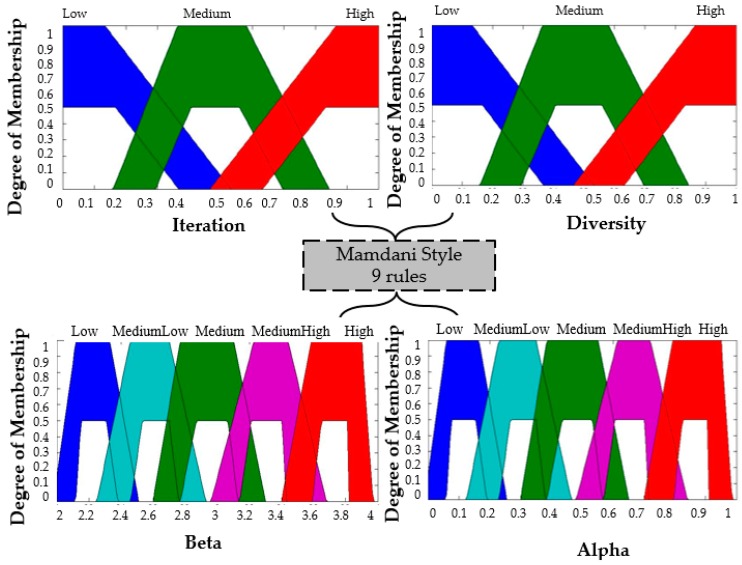
Fuzzy BCO with IT2FLS.

**Figure 13:**
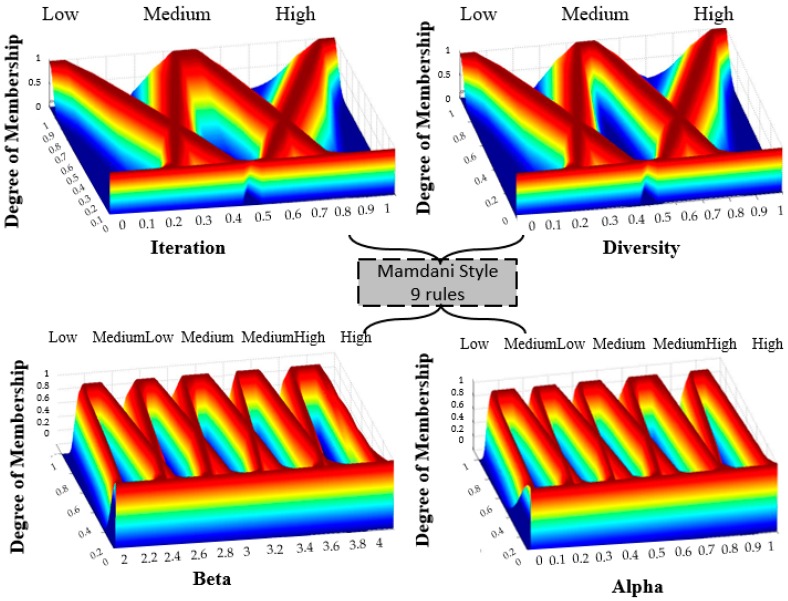
Fuzzy BCO with GT2FLS.

The distribution of the membership functions in the inputs and outputs is realized in a symmetrical way. The design of the input and output variables can be appreciated in Figure 11, Figure 12 and Figure 13 for the Type-1 FLS, Interval Type-2 FLS and Generalized Type-2 FLS, respectively. The fuzzy rules are shown in Table 3.

Various experiments were previously realized in which the idea is to explore the behavior of the BCO algorithm. The interesting factor that was found is that we need to start with high exploration and thus, the proposed methodology is able to analyze better all the search space.

To start the BCO algorithm, the iteration is low and the diversity is low, this is because the initialization of the position of the bees is set randomly in steps 1 of the BCO algorithm. This reasoning that is used for realizing the Rule number 1 which is: “*If Iterations is Low and Diversity is Low then Beta is High and Alpha is Low*”. The high value for beta represents that the bees should realize high exploration and the value low of alpha represents that the bees should have little exploitation in BCO algorithm. On the other hand, when the Iterations are high (last iterations of the BCO algorithm) the bees have a high diversity (bees are separated) and the value of beta is low to obtain low exploration and the value of alpha is high to obtain a better exploitation in the problem. This reasoning is used for realizing Rule number 9, which is: “*If Iterations is High and Diversity is High then Beta is Low and Alpha is High*”.

**Figure 14:**
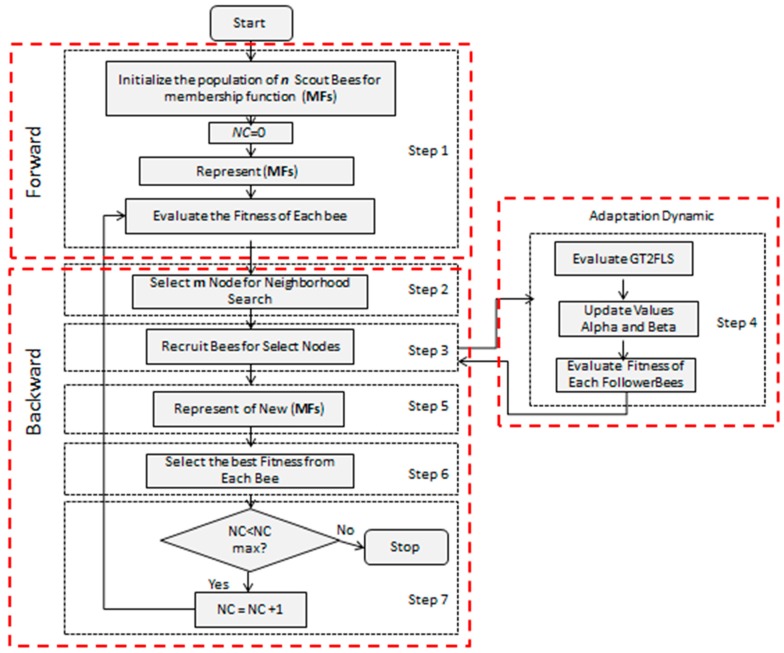
Flowchart of the proposed Fuzzy BCO.

The proposed general flowchart of BCO is illustrated in Figure 14, where “*ScoutBees*” indicates the size of population, “*NC*” represents the number of constructive moves during one forward pass and “*FollowerBees*” represents each bee that explores the possible solutions.

## 6. Results

Experimentation was performed with various external perturbation scenarios. Two specific noise generators are used: band-limited white noise and pulse generated noise. The height of the Power Spectral Density of the band-limited white noise of power is set to (0.5, 1), sample time of (0.5, 1), and delay of 1000; and the amplitude is set to (0.5, 1), period in seconds of 1, pulse width (%) is set to (0.5, 1) and phase delay is set to 1000, respectively, for the pulse generated noise.

We use the problem of controlling a trajectory of an autonomous mobile robot, the test criteria is a series of Performance Indices; where the Integral Square Error (ISE), Integral Absolute Error (IAE), Integral Time Squared Error (ITSE), Integral Time Absolute Error (ITAE) and Root Mean Square Error (RMSE) are used, respectively shown in Equations (23)–(27):

(23) $$ISE=\int_{0}^{\infty }e^{2}\left(t\right)dt$$

(24) $$IAE=\int_{0}^{\infty }\left|e\left(t\right)\right|dt$$

(25) $$ITSE=\int_{0}^{\infty }e^{2}\left(t\right)tdt$$

(26) $$ITAE=\int_{0}^{\infty }\left|e\left(t\right)\right|tdt$$

(27) $$\varepsilon =\sqrt{\frac{1}{N}{\sum_{t\;=\;1}^{N}\left(X_{t}-{\hat{X}}_{t}\right)}^{2}}$$

The BCO algorithm was configured with the parameters listed in Table 4.

The results of the simulations for the problem are presented in Table 5, which shows the errors for each performance index of 30 experiments for the autonomous mobile robot controller using the traditional BCO, and the *alpha* and *beta* values are set to 0.5 and 2.5, respectively. The results in Table 5 were ordered with respect to the minimization of the MSE.

**Figure 15:**
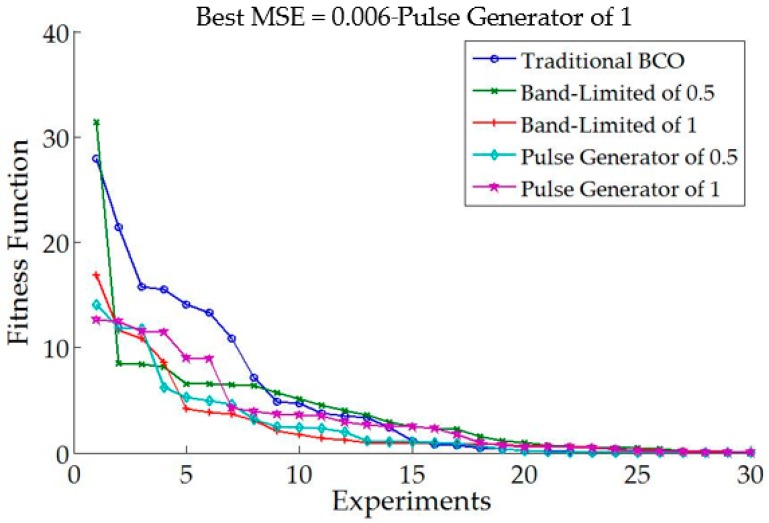
Behavior of the traditional BCO with different perturbation in the model.

In Table 5 can be noted that the best value for the MSE was of 0.002. The simulation results in Table 5 are obtained with the traditional BCO. The main goal of the experiments is to observe the values of alpha and beta in the algorithm to compare with the results of the proposed method, as well as to observe the results with the traditional BCO algorithm with the considered problem. Figure 15 shows the behavior of the MSE when different levels of noise are applied in the traditional BCO.

**Figure 16:**
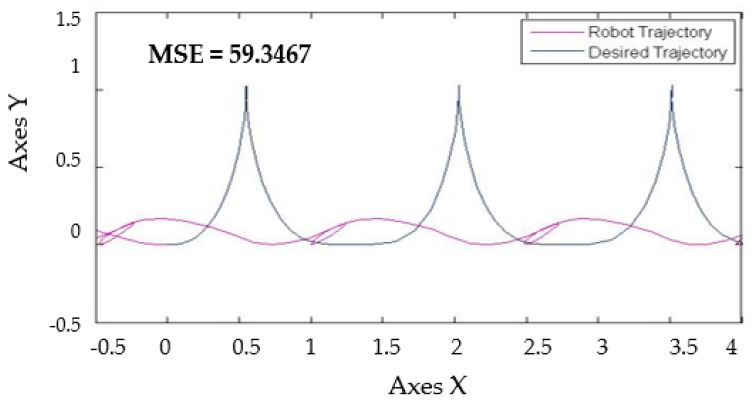
Trajectory in the autonomous mobile robot controller with base FLS with perturbation in the model.

With the base FLS the distribution of membership functions detailed in Figure 9. The behavior in the simulations is shown in Figure 16 with a perturbation in the model of pulse generated with value of 1.

**Figure 17:**
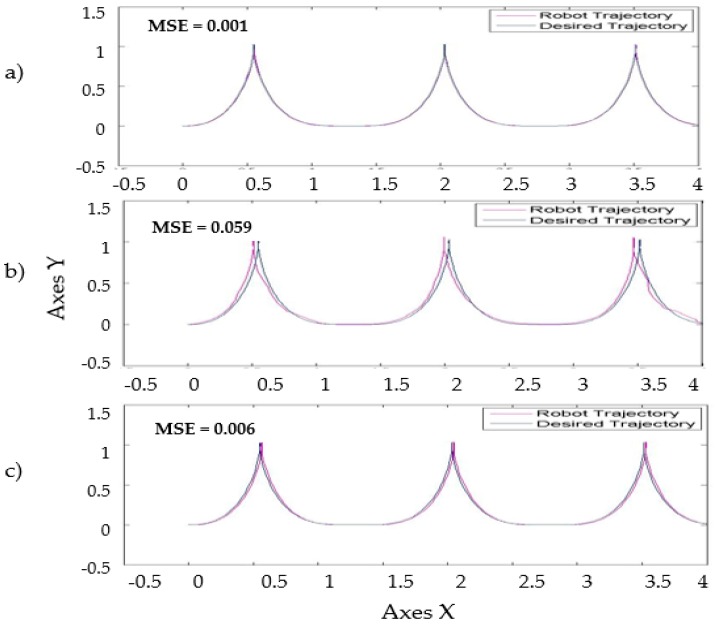
Trajectory in the autonomous mobile robot controller, (**a**)Traditional BCO; (**b**) BCO algorithm applied perturbation called band-limited with value of 1; (**c**) BCO algorithm applied perturbation called pulse generator with value of 1.

Figure 17 shows similar simulation errors when the levels of noise are applied in the model, the stabilization in the trajectory in autonomous mobile robot is shown in Figure 17 with the best MSE using the two types of perturbations in the model with the value of 1 and the traditional BCO.

Table 6 shows the average of 30 experiments, standard deviation (SD), the best and the worst of the simulation errors for the four methods: Traditional BCO, Fuzzy BCO with Type-1 Fuzzy Logic System (FLS), Fuzzy BCO with Interval Type-2 FLS and Fuzzy BCO with Generalized Type-2 FLS without applying perturbation in the model. We used the MSE as the fitness function in the Bee Colony Optimization algorithm.

Table 6 shows that using the traditional method the average MSE was of 5.090 and with Generalized Type-2 FLS was 6.118 using the dynamic adjustment in *alpha* and *beta,* which tells us that if we do not apply perturbation in the model the dynamic adjustment do not provide better results compared to the traditional BCO for the stabilization of the autonomous mobile robot. The *alpha* and *beta* values found by Fuzzy BCO with GT2FLS are 2.601 and 0.467, respectively. We applied perturbation in the model, and this is a way of analyzing uncertainty in the fuzzy sets. Table 7 shows results with the pulse generator with a value of 0.5 for each methodology used.

Table 7 shows that using the traditional method with perturbation in the model the average MSE was 2.601 and with Generalized Type-2 FLS was 2.467 using the dynamic adjustment in alpha and beta values, which tells us that if we apply perturbation in the model the dynamic adjustment produces better results compared to the traditional BCO for the stabilization of the autonomous mobile robot. The minimum value of MSE was 0.001 for the Fuzzy BCO with Type-1 FLS. The best alpha and beta values found by GT2FLS are 2.601 and 0.467, respectively.

**Figure 18:**
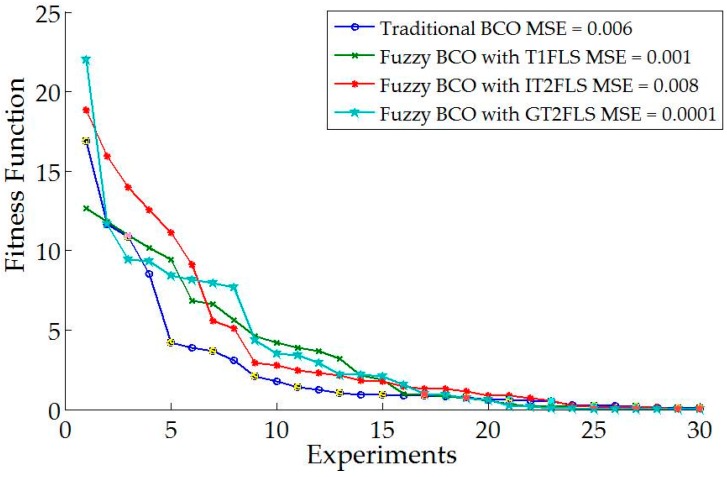
Comparative results of each method with different perturbation in the model.

Figure 18 shows comparative results when applying a pulse generator with a value of 1 in the model. The MSE is shown for comparison.

Figure 18 shows that the best error is found by GT2FLS with a value of 0.0001. It is important to mention that GT2FLS starts with a low error, but the stabilization in the trajectory of the autonomous mobile robot is high compared to other methods.

**Figure 19:**
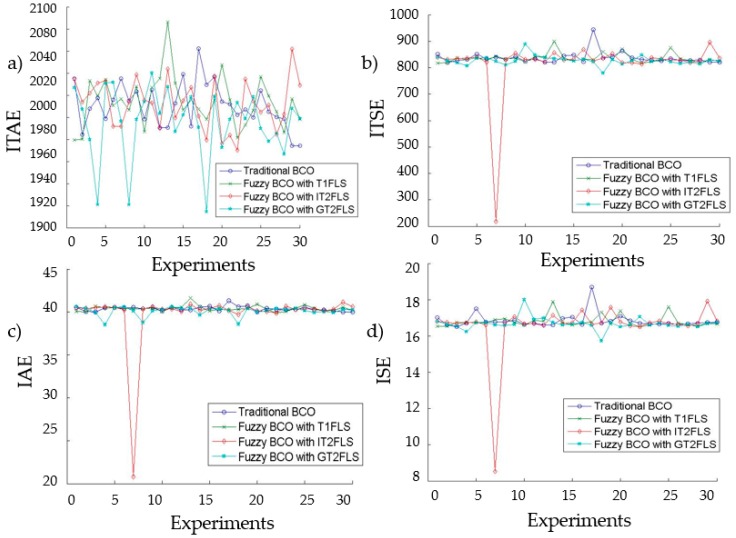
Behavior of various performance indices in relation to band-limited perturbations with value of 1 presents the system, when Fuzzy Bee Colony Optimization-Type-1 Fuzzy Logic System, FBCO-Interval Type-2 Fuzzy Logic System and FBCO-Generalized Type-2 Fuzzy Logic System are used. (**a**) ITAE; (**b**) ITSE; (**c**) IAE; and (**d**) ISE.

As an example of the relation between noise and the FLS performance, Figure 19 shows these relations for each type of performance index used, where in all accounts FBCO with GT2FLS is somewhat better with respect to Fuzzy BCO with IT2FLS and then to Fuzzy BCO with T1FLS.

Two scenarios in the experiments were changed, the first is to observe the behavior of the Interval Type-2 FLS, a reduction in the size of the Footprint Uncertainty (FOU) to a value of 0.5 was realized. In experiments previously performed, the value of FOU was set to 0.9. The second is to minimize the value of the FOU to 0.5 and also, increase the value of the volume (depth) of a generalized membership function, the value set in previous experiments was of 0.5, for these experiments we change it to 1, which indicates that we have more secondary functions memberships to evaluate with the Generalized Type-2 FLS. The averages of 30 experiments are presented in Table 8.

**Figure 20:**
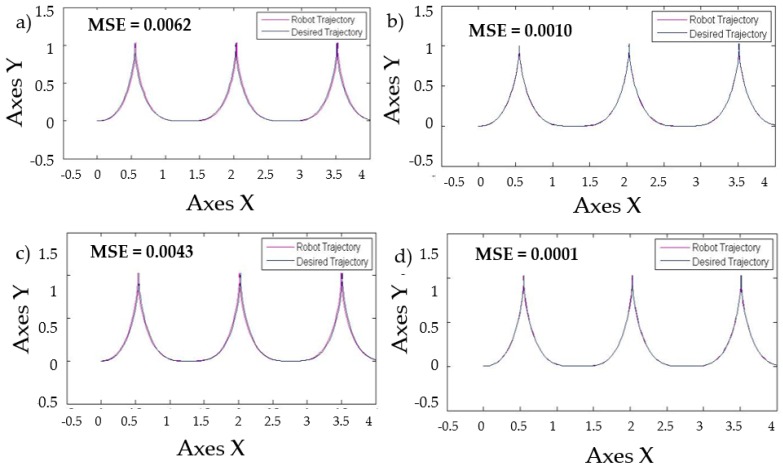
Behavior of trajectory for each method with pulse generator perturbations with value of 1. (**a**) Traditional BCO; (**b**) Fuzzy BCO with Type-1 FLS; (**c**) Fuzzy BCO with IT2FLS; and (**d**) Fuzzy BCO with GT2FLS.

Table 8 shows that when levels of noise are used in the model, the stabilization of the autonomous mobile robot is better with the Fuzzy BCO with GT2FLS compared to the traditional BCO. The standard deviation is smaller, which indicates that the results are similar. The best MSE error found was by FBCO-GT2FLS with a value of 0.0001. The behavior of the trajectory of the autonomous mobile robot is shown in Figure 20 with perturbation (pulse generator of 1) in the model. The Best MSE is also shown in Figure 20.

**Figure 21:**
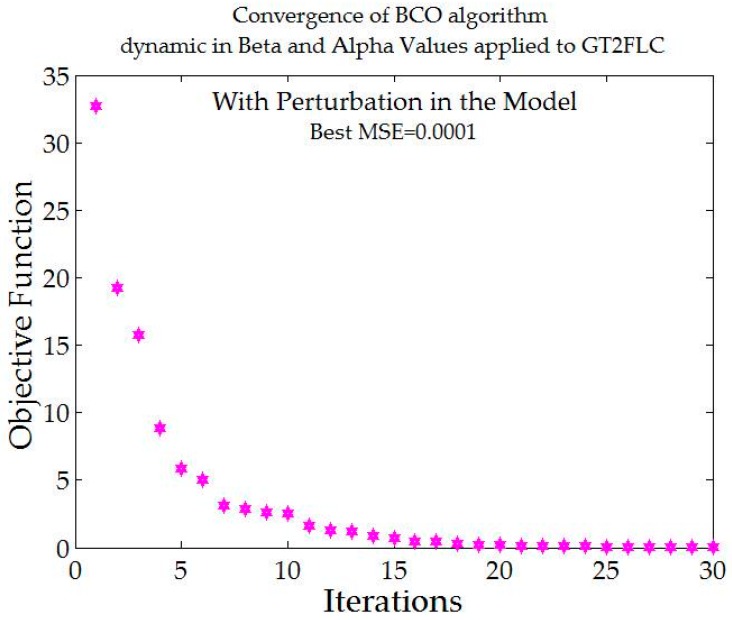
Convergence of the MSE error with adjustment in alpha and beta values.

The convergence of the Fuzzy BCO with Generalized Type-2 FLS with dynamic alpha and beta values is shown in Figure 21.

**Figure 22:**
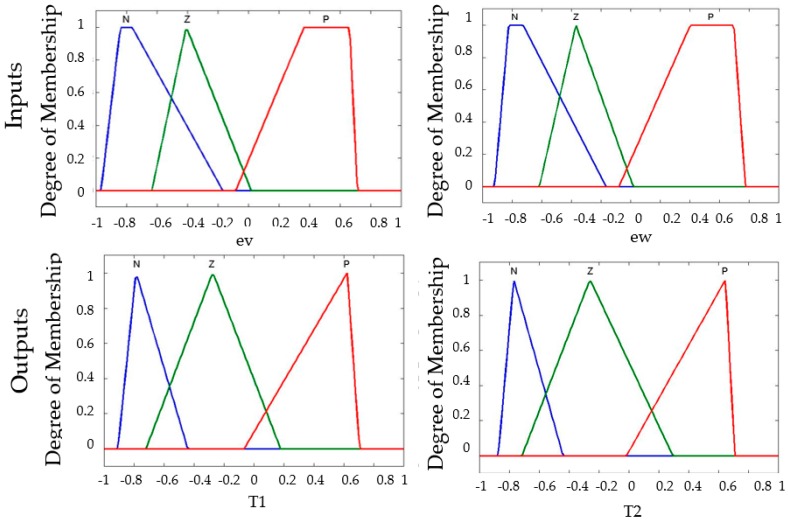
Best Distribution of membership function found by FBCO with Generalized Type-2 FLS.

**Figure 23:**
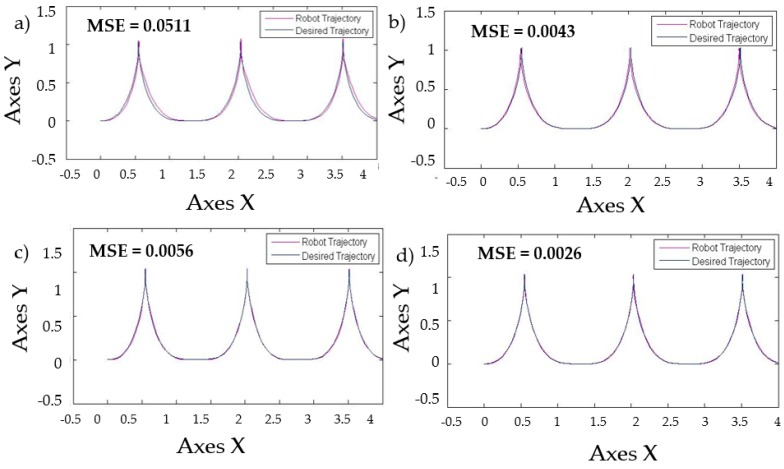
Behavior of trajectory for each method applying perturbation in the model. (**a**) Traditional BCO; (**b**) Fuzzy BCO with Type-1 FLS; (**c**) Fuzzy BCO with IT2FLS; and (**d**) Fuzzy BCO with GT2FLS.

The best distribution in membership functions found by FBCO with Generalized Type-2 FLS is shown in Figure 22. With the objective to observe the performance that a Generalized Type-2 FLS has with respect to IT2FLS, T1FLS and Traditional BCO. The best FLS found by each method using simulations without perturbation in the model that was selected. We added more perturbation such as; a pulse generator of amplitude of 5 and pulse width of 5. The result of trajectory in the autonomous mobile robot is shown in Figure 23.

**Figure 24:**
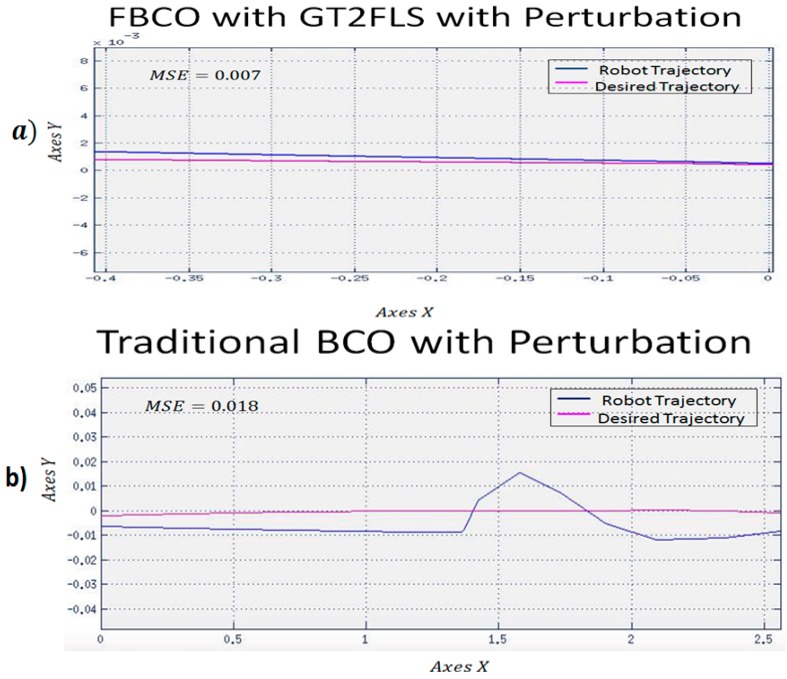
Behavior of the second trajectory for the proposed method applying perturbation in the model. (**a**) FBCO with GT2FLS (**b**) Traditional BCO algorithm.

To observe the efficiency of proposed method, a different trajectory is shown in Figure 24, where we have shown the best experiment with FBCO with GT2FLS and the best experiment of the Traditional BCO used the perturbation pulse generator with value of 0.5 (see Table 7).

With a linear trajectory, in Figure 24 the proposed method (a) the pink line (robot trajectory) is closest to the yellow line (desired trajectory). This simulation is reflected with the MSE that the FBCO with GT2FLS found with a value of 0.007 compared to the Traditional BCO (Figure 24b) that had the best MSE of 0.018 with perturbations in the model.

## 7. Discussion

Every problem that has been analyzed required the optimal parameters for an optimization algorithm. For this reason is necessary to realize several experiments to meet these parameters. In this work, we realized a study to determine how the alpha and beta values affect the performance of the BCO algorithm applied in the fuzzy controller, and to obtain whit this design of the fuzzy rules to the GT2FLS.

Based on the experiments in Section 5, we analyzed the results; the Traditional BCO algorithm is a good technique for the optimization and design of the fuzzy controller, because the behavior of the trajectory in the autonomous mobile robot without applying perturbation in the model is good (see Table 5), but when we increased the levels of noise, the stabilization in the model is better with the adjustment dynamic of parameters using GTL2FLS (see Figure 23), this is because the uncertainty is better handle and the perturbations the level of noise is minimized with GT2FLS.

When the size of the FOU in the Interval Type-2 FLS is increasing (see Table 8) the perturbation is better analyzed, the best MSE found was of 0.004 compared to the Traditional BCO that was of 0.008. It is important to mention that when the size of Volume in GT2FLS is increasing (see Table 8) to allow evaluate more secondary membership functions and the standard deviation is smaller with a value of 3.32 with perturbation in the model compared to 7.27 with Traditional BCO; this determines that all the results found by GT2FLS are similar.

Future work in this research consists in applying this technique to find the optimal values in the *alpha* and *beta* parameters for the optimization the Interval Type-2 Fuzzy Logic Controller and Benchmark Functions with the Fuzzy BCO algorithm, and thus able to observe that the optimal parameters found with this method are adapted to any optimization problem.

## 8. Conclusions

In this paper, we conclude that the BCO algorithm is a good optimization technique for design and stabilization of fuzzy controllers. The process of dynamically adjusting parameters of an optimization method (in this case the Bee Colony Optimization algorithm), can improve the quality of results and increasing the diversity of solutions to a problem. Three fuzzy systems were designed for the adjustment the parameters for BCO algorithm, T1FLS, IT2FLS and GT2FLS. These proposed methods show the quality of the results better that the Traditional BCO when the design of the parameters in a fuzzy logic system for an autonomous mobile robot in simulation allow the stabilization of the trayectory and minimization of the error efficiently.

Experiments were performed with the proposed method to find the optimal alpha and beta values in the parameters of BCO algorithm, and a comparison was made between the method of traditional bee colony optimization and the proposed methods, i.e., with the three fuzzy systems for parameters adjustment.

The BCO algorithm was implemented to find the optimal distribution of parameters in the design of the fuzzy controller, especially in controlling the trajectory in an autonomous mobile robot, thus being able to demonstrate the efficiency of the Fuzzy BCO algorithm as a technique to improve the performance in fuzzy controllers. By comparing the proposed methods and the Traditional BCO algorithm, in the design of fuzzy logic systems applied to fuzzy control it was found that based on the experiments, it was possible to develop a method for dynamically adjusting the alpha and beta parameters of the BCO through the three fuzzy logic systems. And in this way improving the results compared with the simple BCO method.

The performance of each fuzzy system, used in this research was observed in the results, when IT2FLS is increased the FOU size and when GT2FLS is increasing the volume size.

## Figures and Tables

**Table 1:** Fuzzy Rules used by the Fuzzy Controller.

*# Rules*	*Input 1*	*Input 2*	*Output 1*	*Output 2*
	*ev*	*ew*	*T1*	*T2*
1	N	N	N	N
2	N	Z	N	Z
3	N	P	N	P
4	Z	N	Z	N
5	Z	Z	Z	Z
6	Z	P	Z	P
7	P	N	P	N
8	P	Z	P	Z
9	P	P	P	P

Angular velocity (*ev*) and negative and linear velocity (*ew*).

**Table 2:** Basic Steps of the BCO Algorithm.

Pseudocode of BCO
Initialization: an empty solution is assigned to every bee; For every bee: //the forward pass (a) Set k = 1; //counter for constructive moves in the forward pass; (b) Evaluate all possible constructive moves; (c) According to evaluation, choose on move using the roulette wheel; (d) k = k + 1; if k ≤ NC goto step b. All bees are back to the hive; //backward pass starts. Evaluate (partial) objective function value for each bee; Every bee decide randomly whether to continue its own exploration and become a recruiter, or to become a follower; For every follower, choose a new solution from recruiters by the roulette wheel; If solutions are not completed goto step 2; Evaluate all solutions and find the best one; If stopping condition is not met goto step 2; Output the best solution found.

**Table 3:** Rules for the Fuzzy BCO with dynamic adaptation of the beta and alpha parameter values.

# Rules	Input 1	Input 2	Output	Output
	Iteration	Diversity	Beta	Alpha
1	Low	Low	High	Low
2	Low	Medium	MediumHigh	Medium
3	Low	High	MediumHigh	MediumLow
4	Medium	Low	MediumHigh	MediumLow
5	Medium	Medium	Medium	Medium
6	Medium	High	MediumLow	MediumHigh
7	High	Low	Medium	High
8	High	Medium	MediumLow	MediumHigh
9	High	High	Low	High

**Table 4:** Configuration parameters of the BCO algorithm.

Parameter	Values
Population	50
Employed Bee	30
Iterations	30
Alpha	Dynamic
Beta	Dynamic

**Table 5:** Simulation errors with the traditional BCO algorithm without a level of noise.

No.	Performance Index						
	ITAE	ITSE	IAE	ISE	MSE	RMSE	TIME (Minutes)
01	2000.504	800.392	40.048	16.051	**0.002**	0.841	5:08
02	1943.814	777.649	39.342	15.819	0.008	0.096	4:10
03	1934.216	773.967	39.258	15.758	0.019	1.866	6:44
04	1990.523	796.011	39.876	16.023	0.020	0.801	4:23
05	1950.637	780.445	39.574	15.893	0.030	0.369	5:56
06	1964.311	785.927	39.482	15.884	0.043	0.175	5:03
07	1966.415	786.696	39.695	15.932	0.049	0.420	5:27
08	1969.791	788.537	39.582	16.039	0.074	0.236	5:36
09	1996.253	798.623	39.893	16.002	0.111	0.138	12:20
10	1985.638	794.689	39.821	16.101	0.125	1.887	5:25
11	1985.033	794.243	39.813	16.008	0.170	2.437	4:42
12	1990.986	823.919	39.946	16.462	0.360	5.023	4:27
13	1910.116	764.161	39.012	15.650	0.452	0.452	4:14
14	2002.708	802.336	40.055	16.056	0.758	0.796	7:28
15	1982.331	802.564	39.941	16.168	0.764	4.370	4:45
16	1964.583	786.031	39.489	15.876	1.108	1.722	4:31
17	1933.323	773.438	39.277	15.784	2.412	14.823	6:07
18	1993.029	797.686	39.944	15.988	3.375	5.093	5:20
19	1942.634	777.156	39.332	15.791	3.538	3.765	5:46
20	1991.067	796.604	39.833	15.991	3.735	4.025	4:26
21	1921.713	768.716	39.246	15.724	4.688	5.050	5:20
22	1973.170	789.419	39.682	15.963	4.843	7.021	6:11
23	2000.093	800.126	40.012	16.054	7.117	15.322	5:30
24	1963.144	785.513	39.654	15.899	10.831	14.964	6:07
25	2025.914	834.576	40.595	16.735	13.326	23.153	5:22
26	1872.314	749.050	38.658	15.531	14.049	17.266	6:36
27	1989.600	796.013	39.747	15.977	15.535	18.916	7:10
28	1999.003	845.801	40.053	16.996	15.741	15.750	5:22
29	1935.925	776.054	39.231	15.813	21.458	21.458	6:52
30	1943.784	777.582	39.409	15.848	27.951	37.567	7:21
**Average**	**1967.419**	**790.797**	**39.650**	**15.994**	**5.090**	**7.527**	

**Table 6:** Simulation results without perturbation in the model.

Performance Index		Methods			
		Traditional BCO	Fuzzy BCO with Type-1 FLS	Fuzzy BCO with Interval Type-2 FLS	Fuzzy BCO with Generalized Type-2 FLS
ITAE		1967.419	1834.635	1890.023	1918.058
ITSE		790.797	744.277	773.802	779.201
IAE		39.650	37.054	38.416	38.918
ISE		15.994	15.098	15.770	15.887
MSE		5.090	2.599	4.059	6.118
RMSE		7.527	5.416	7.385	8.352
**MSE**	**Standard Deviation**	7.276	3.322	5.965	13.000
	**Best**	0.002	0.004	0.006	0.003
	**Worst**	27.951	13.132	24.411	66.507
**Beta**		2.5 (Fixed)	3.074	2.599	2.601
**Alpha**		0.5 (Fixed)	0.661	0.466	0.467

**Table 7:** Simulation results with pulse generator perturbation in the model.

Performance Index		Methods			
		Traditional BCO	Fuzzy BCO with Type-1 FLS	Fuzzy BCO with Interval Type-2 FLS	Fuzzy BCO with Generalized Type-2 FLS
ITAE		1974.966	1951.805	1893.691	1895.279
ITSE		795.631	785.731	762.295	759.013
IAE		39.340	37.545	38.242	38.269
ISE		16.110	15.945	15.456	15.380
MSE		2.601	3.490	3.361	2.467
RMSE		4.460	7.478	6.750	8.149
**MSE**	**Standard Deviation**	3.764	4.875	4.122	8.313
	**Best**	0.018	0.001	0.034	0.007
	**Worst**	14.048	23.393	4.122	40.853
**Beta**		2.5(Fixed)	2.992	2.812	2.601
**Alpha**		0.5(Fixed)	0.784	0.494	0.467

**Table 8:** Simulation results minimizing Footprint Uncertainty (FOU) and increasing volume.

Method	Level of Noise	Performance Index								
		ITAE	ITSE	IAE	ISE	MSE	RMSE	SD	Best MSE	Worst MSE
Traditional BCO	N/A	1967.41	790.79	39.65	15.99	5.09	7.52	7.27	0.002	27.95
	Band-Limited = 1	1878.92	782.34	37.81	15.76	2.63	5.60	3.99	0.059	16.89
	Pulse Generator = 1	1965.27	972.43	39.56	16.02	3.91	6.49	5.21	0.008	18.65
FBCO with IT2FLS	N/A	1893.69	762.29	38.24	15.45	3.36	6.75	4.12	0.03	15.43
	Band-Limited = 1	1825.72	762.65	39.99	15.45	3.18	6.59	3.79	0.02	17.17
	Pulse Generator = 1	1961.68	788.96	39.58	15.98	4.04	7.42	5.78	0.004	26.49
FBCO with GT2FLS	N/A	1834.63	744.27	37.05	15.09	2.59	5.14	3.32	0.004	13.13
	Band-Limited = 1	1935.24	806.40	38.85	16.21	2.95	5.11	3.67	0.024	15.77
	Pulse Generator = 1	1973.42	799.61	39.75	16.16	3.69	5.62	**4.89**	**0.001**	21.99

## Abbreviations

BCO	Bee Colony Optimization
T1FLS	Type-1 Fuzzy Logic System
IT2FLS	Interval Type-2 Fuzzy Logic System
GT2FLS	Generalized Type-2 Fuzzy Logic System
FLC	Fuzzy Logic Controller.
MSE	Mean Square Error
RMSE	Root Mean Square Error
ITAE	Integrated Time Absolute Error
ITSE	Integrated Time Square Error
IAE	Integrated Absolute Error
ISE	Integrated Square Error
SD	Standard Desviation
